# Do nonphysical punishments reduce antisocial behavior more than spanking? a comparison using the strongest previous causal evidence against spanking

**DOI:** 10.1186/1471-2431-10-10

**Published:** 2010-02-22

**Authors:** Robert E Larzelere, Ronald B Cox, Gail L Smith

**Affiliations:** 1 Department of Human Development and Family Science, 233 HES Bldg., Oklahoma State University, Stillwater, OK 74078, USA; 2 Girls and Boys Town, Boys Town, NE 68010, USA

## Abstract

**Background:**

The strongest causal evidence that customary spanking increases antisocial behavior is based on prospective studies that control statistically for initial antisocial differences. None of those studies have investigated alternative disciplinary tactics that parents could use instead of spanking, however. Further, the small effects in those studies could be artifactual due to residual confounding, reflecting child effects on the frequency of all disciplinary tactics. This study re-analyzes the strongest causal evidence against customary spanking and uses these same methods to determine whether alternative disciplinary tactics are more effective in reducing antisocial behavior.

**Methods:**

This study re-analyzed a study by Straus et al.[1] on spanking and antisocial behavior using a sample of 785 children who were 6 to 9 years old in the 1988 cohort of the American National Longitudinal Survey of Youth. The comprehensiveness and reliability of the covariate measure of initial antisocial behavior were varied to test for residual confounding. All analyses were repeated for grounding, privilege removal, and sending children to their room, and for psychotherapy. To account for covarying use of disciplinary tactics, the analyses were redone first for the 73% who had reported using at least one discipline tactic and second by controlling for usage of other disciplinary tactics and psychotherapy.

**Results:**

The apparently adverse effect of spanking on antisocial behavior was replicated using the original trichotomous covariate for initial antisocial behavior. A similar pattern of adverse effects was shown for grounding and psychotherapy and partially for the other two disciplinary tactics. All of these effects became non-significant after controlling for latent comprehensive measures of externalizing behavior problems.

**Conclusions:**

These results are consistent with residual confounding, a statistical artifact that makes all corrective actions by parents and psychologists appear to increase children's antisocial behavior due to child effects on parents. Improved research methods are needed to discriminate between effective vs. counterproductive implementations of disciplinary tactics. How and when disciplinary tactics are used may be more important than which type of tactic is used.

## Background

Although physical punishment is used by as many as 94% of parents in some countries,[2] a growing number of countries have banned its use[3]. Some American pediatricians oppose all use of corporal punishment, whereas others think there are situations where nonabusive spanking should remain a disciplinary option for parents[4]. The author of one major literature review opposes all spanking,[5,6] whereas five other recent literature reviews have made less absolute conclusions [7-11]. In either case, pediatricians need research evidence comparing the effects of alternative disciplinary tactics with spanking to advise parents about which tactics to use instead of spanking. To provide that information, the present study is one of the first causally relevant longitudinal studies to compare the child outcomes of alternative disciplinary tactics with those of customary spanking. Specifically, it attempts to duplicate the strongest causal evidence against customary spanking to date[1] and investigates which of three types of nonphysical punishment are more effective at reducing antisocial behavior than is customary spanking. Second, we investigate whether or not that evidence is a statistical artifact due to residual confounding from child effects on parents.

In the only scientific consensus conference on corporal punishment, co-sponsored by the American Academy of Pediatrics,[12] both critics[7,13,14] and supporters[15,16] of spanking bans acknowledged the weakness of the relevant scientific evidence. The conference consensus statements thus included appropriate cautions about corporal punishment, but fell short of a blanket opposition to all spanking[17].

Since the literature review published for that conference,[7] there have been at least five published literature reviews with a diversity of conclusions[5,8-11]. For example, Gershoff's[5] meta-analysis supported an anti-spanking perspective;[6] whereas Larzelere and Kuhn's meta-analysis[9] concluded that child outcomes of physical punishment were more adverse than those of alternative disciplinary tactics only for overly severe or predominant use of physical punishment.

Gershoff's[5] meta-analysis concluded that physical punishment was associated with 10 adverse outcomes, whereas immediate compliance was the only benefit associated with spanking. The 10 adverse mean effect sizes, however, were based on cross-sectional, retrospective, or prospective correlations for 60%, 26%, and 14% of the supporting studies, respectively. It is well-known that meta-analyses of correlational evidence can produce precise but spurious results[18,19]. As has been shown elsewhere,[20] selection biases cause even prospective correlations to be biased against most corrective actions, whether implemented by parents or professionals. For example, hospitalization is associated with about a 30-fold increased risk of dying in Medicare patients, and psychotherapy is associated with a median 14-fold increased risk of suicide, compared to matched-age groups not receiving (or needing) those corrective actions. Just as the severity of the presenting condition accounts for the prospective longitudinal correlations between hospitalization and mortality and between psychotherapy and suicide, the severity of oppositional behavior in children may lead parents to use all disciplinary enforcements more often, not just spanking[21].

To adjust for this selection bias, the meta-analysis by Larzelere and Kuhn[9] compared effect sizes for physical punishment and alternative tactics investigated in the same studies. The outcomes of physical punishment compared unfavorably with alternative disciplinary tactics only when it was the *primary *disciplinary method or was too severe (such as beating up a child or striking the face or head). The outcomes of customary spanking were neither better nor worse than for any alternative tactic, except for one study in which spanking reduced drug abuse more than nonphysical punishment[22]. Customary spanking was defined as ordinary usage, without any emphasis on how severely it was used. Larzelere and Kuhn also identified an optimal type of nonabusive back-up spanking, used when a child responds defiantly to milder disciplinary tactics such as time out (based mostly on research on 2- to 6-year-olds). Under these conditions, back-up spanking led to less noncompliance or antisocial behavior than 10 of 13 alternative disciplinary tactics and produced outcomes equivalent to the other three tactics. The nine relevant studies included the only four randomized clinical trials of spanking, which yielded the strongest causal evidence about spanking in the scientific literature, albeit limited to enforcing compliance with time out in clinically defiant 2- to 6-year-olds [23-26]. Compliance with time out is a crucial component for effective implementation of most evidence-based psychosocial treatments for Oppositional Defiance Disorder, Conduct Disorder, and ADHD in young children[27,28].

A second development since 1996 is new evidence that customary spanking predicts greater subsequent antisocial behavior longitudinally after controlling statistically for initial differences in antisocial behavior. A seminal study by Straus and his colleagues in 1997[1] provided the first evidence against customary spanking based on stronger causal evidence than unadjusted correlations. This improved causal evidence has led some to conclude that any use of spanking is invariably detrimental and should be opposed by all professionals[29].

We located 14 longitudinal studies that investigated whether physical punishment of children younger than 13 years old predicted subsequent antisocial behavior or aggression after controlling statistically for initial levels of those outcome variables. Seven of them lumped together nonabusive spanking with more severe forms of punishment, such as shaking, hitting with an object, or name-calling [30-36]. The remaining seven studies showed non-significant,[21,37,38] small,[1,39,40] or mixed effects[41] of customary spanking on subsequent antisocial behavior or aggression. The small significant effects were found only for non-Hispanic European-Americans or in samples dominated by that group, with effect sizes of β = .05,[39] .06,[40] and .07[1]. Significantly adverse outcomes emerged only in studies in which mothers reported spanking frequency in the past week. The studies also used maternal reports for the outcome variable except for Gunnoe and Mariner,[41] which found contrasting effects for different subgroups. With a distinct source of information for the child outcome variable (i.e., child report), Gunnoe and Mariner found that customary spanking significantly *reduced *aggression in the following subgroups: all 4- to 7-year-olds, all African-Americans, and all girls, although spanking increased aggression in all 8- to 11-year-olds and in all European-Americans. They also replicated the usual small adverse effect of customary spanking on antisocial behavior when relying solely on parental report[41].

In sum, the correlational evidence against spanking that was considered weak evidence by most participants in the 1996 scientific consensus conference was replicated in Gershoff's meta-analysis[5]. Since then, the causal evidence against spanking has been strengthened by seven studies that have found small, sometimes significant adverse effects of customary (non-severe) spanking on subsequent antisocial behavior, after controlling statistically for pre-existing antisocial scores. On the other hand, Larzelere and Kuhn's meta-analysis[9] found that child outcomes of physical punishment were more adverse than those for alternative disciplinary tactics only when physical punishment was overly severe or the predominant disciplinary tactic. No published study has compared the outcomes of any alternative disciplinary tactic with those of customary spanking in statistically controlled longitudinal analyses, a gap addressed by this study.

In addition to investigating the ability of alternative disciplinary tactics to reduce antisocial behavior, this study will investigate whether the small adverse effects attributed to spanking in statistically controlled analyses could be due to residual confounding[42]. In statistically controlled analyses, residual confounding explained why the summer Head Start program appeared to be detrimental according to a major early evaluation study[43,44]. Statistical controls yield unbiased estimates of causal effects only when the process of selecting recipients for a corrective action is measured comprehensively[45] and without measurement error[46,47]. Statistically controlled studies with fallible measures of the selection process only reduce the artifactual selection bias confounded with corrective actions[20,48]. Accordingly, epidemiologists recognize that residual confounding remains when confounds are only partially controlled for statistically[42].

If the association between the frequency of spanking and subsequent antisocial behavior is due to child differences in initial levels of oppositional behavior, it follows that all disciplinary enforcements should show a similar association with antisocial behavior. This result would be consistent with Larzelere and Kuhn's meta-analysis that found no differences in effect sizes in comparisons between customary spanking and alternative disciplinary tactics[9]. No statistically controlled longitudinal study of customary spanking has also investigated alternative disciplinary tactics that parents could use instead of spanking. This is therefore the first study to our knowledge that compares antisocial behavior outcomes of alternative disciplinary tactics vs. customary spanking after controlling statistically for pre-existing differences on antisocial behavior.

### Analytic Plan

To compare the effects of three types of nonphysical punishment with the effects of spanking on subsequent antisocial behavior, we duplicated the study with the strongest causal evidence against customary spanking as closely as possible. Straus et al.[1] was selected because it has reported the largest effect size associating spanking frequency with subsequent antisocial behavior. Their somewhat larger effect size might be partly explained because they chose to feature the cohort (out of five possible cohorts) with the largest longitudinal correlation between Wave-1 spanking and Wave-2 antisocial behavior (*r *= .29, compared to a mean of *r *= .22 in the other four cohorts in their Table 1, p. 764) [1]. By duplicating the strongest causal evidence against customary spanking, our study increases the likelihood of finding disciplinary alternatives with better child outcomes than spanking.

If alternative disciplinary tactics show the same adverse associations with subsequent antisocial behavior as shown by spanking, however, the small effect sizes could be due to residual confounding. To test that possibility, additional analyses determined whether the adverse outcomes remained significant after improving the measure of pre-existing differences. Complete removal of the confound of oppositional behavior in children requires that oppositional behavior be measured comprehensively and without measurement error[47]. Therefore, it follows that if the causal link between disciplinary punishments and antisocial behavior is artifactual due to residual confounding, then the adverse effects should become smaller and non-significant with improved measures of pre-existing antisocial behavior. Improvement in comprehensiveness will be evaluated in this study by comparing the trichotomous covariate used in Straus et al.[1] with a continuous measure of the 6-item antisocial behavior scale and a 16-item measure of externalizing behavior problems. Structural equation modeling will also be used because it minimizes measurement error in the measure of pre-existing externalizing behavior problems[49].

A final set of analyses will determine whether disciplinary tactics predict simple change scores in externalizing behavior problems in the same direction that they predict residualized change scores in the above analyses. Simulation studies have shown that most analyses of residualized change scores remain biased against corrective actions even though they control statistically for pre-existing differences[44,50]. In contrast, analyses of simple gain scores are biased in favor of corrective interventions due to regression toward the mean in the only known simulation study[51]. Several prominent methodologists have recommended analyses of simple gain scores instead of residualized gain scores for many situations [52-54]. A recent study of Canadian longitudinal data showed that analyses of residualized gain scores were biased against corrective actions implemented by both parents and professionals, whereas analyses of simple gain scores were biased in favor of those corrective actions in the same data[48]. When confounding factors are completely corrected for, however, analyses of residualized gain scores and simple gain scores agree with each other[55,56].

Finally, to evaluate the success of the covariate adjustments in this study, the results will include outcomes of psychotherapy for comparative purposes. If the results are due to residual confounding, the pattern of results should be similar for corrective actions by professionals as well as corrective disciplinary actions by parents.

Because some parents will have used no disciplinary tactics in the past week and others will have used multiple tactics, we also repeated all the above analyses with two additional variations[57]. The first variation repeated the analyses for the subset of families that reported at least one disciplinary tactic during the past week. The second variation included all disciplinary tactics and psychotherapy in the same analyses of the full sample, thereby controlling for each other.

In summary, we re-analyzed the strongest causal evidence against customary spanking,[1] using the same National Longitudinal Survey of Youth (NLSY) cohort to investigate the apparent effect of spanking on subsequent antisocial behavior. Second, we repeated those analyses with each of three alternative disciplinary tactics: grounding, privilege removal, and sending children to their room. Third, those analyses were repeated while varying the adequacy of the covariate used for initial antisocial behavior. Covariates include a dichotomous measure, the trichotomous measure used by Straus et al.,[1] a continuous measure of the 6-item antisocial subscale, and a continuous measure of a 16-item scale of externalizing problems. Fourth, structural equation modeling was used to control for a latent factor of externalizing problems, thereby minimizing measurement error in the covariate. Fifth, we predicted simple changes in latent externalizing behavior problems, which should reverse the direction of the selection bias due to child differences if the effects are due to residual confounding associated with initial antisocial behavior. Sixth, we implemented all analyses for psychotherapy. Seventh, we repeated all these analyses for the subsample receiving any disciplinary tactics. Finally, we repeated the analyses in the full sample with all disciplinary tactics and psychotherapy as simultaneous predictors of antisocial behavior.

## Methods

### Participants

The participants were the mothers of children between the ages of 6 and 9 during the 1988 wave of the Child Supplement of the National Longitudinal Survey of Youth (NLSY) [58] who had valid scores on all relevant variables in 1988 and 1990. The mothers in the NLSY came from a nationally representative sample of 6,283 females aged 14 to 21 years old in 1979, with an over-representation of African-Americans, Hispanics, poor Whites, and military personnel. By 1988, the young women ranged in age from 23 to 30; thus the children in this study were born when they were between 14 and 24 years old (*M *= 20.0). The sample therefore consists of children of young mothers. Most military mothers and the over-sampled poor Whites were dropped from the longitudinal study, leaving 4,941 eligible in 1990. Of these, 4,510 (91%) were interviewed in 1990, including some information on 5,803 of the 5,949 children living with the 68% of the sample who were mothers by that time. Of these children, 1,512 were between 8 and 11 years old in 1990[58]. Based on Straus et al.,[1] 996 of these children had valid data on spanking in 1988 and on antisocial behavior in 1990. Some of those were missing data on other variables used as 1988 covariates or represented multiple children from the same families. After eliminating those cases, Straus et al. had 807 mother-child pairs in their final sample[1].

In order to re-analyze Straus et al. [1] as closely as possible, we duplicated their final sample size of 807 families with valid scores on all the variables in their analyses. Following their procedure, we randomly selected one child from families with multiple eligible children. We drew 10 random samples from the multiple-child families to match their total sample size of 807. We then dropped any cases with missing data on either grounding, privilege removal, or sending children to their room, so that comparisons among these disciplinary enforcements would be based on identical cases. This yielded a final sample size of 785 for the primary analyses. We ranked the effect sizes of spanking and of the three alternative disciplinary tactics in predicting subsequent antisocial behavior in the 10 random samples, controlling for all variables in the original study. We then selected the sample for which the effect of spanking was above its median effect size and most closely approximated the average ranking of the three alternative tactics. The selected sample had the fourth strongest effect for spanking among the 10 samples (*p *= .010), and the rankings for grounding (4th), privilege removal (5th), and sending to their room (1^st^) averaged 3.3. For all 10 samples, the median *F *values were *F *(3, 741) = 3.63, *p *= .013 for spanking; *F *(3, 741) = 1.95, *p *= .12 for grounding; *F *(3, 741) = 1.80, *p *= .15 for privilege removal; and *F *(3, 741) = 2.49, *p *= .06 for sending children to their room. This study was exempt from review by a research ethics committee because it used publicly available data with no identifying information.

### Measures

We used the same variables from the Child Supplement of the National Longitudinal Study of Youth (NLSY) as reported by Straus et al[1]. The primary outcome was the 1990 NLSY Antisocial subscale from the Behavior Problems Index[58]. These maternal-report items included the extent to which the child "cheats or tells lies," "bullies or is cruel or mean to others," "does not feel sorry after misbehavior," "breaks things deliberately," "is disobedient at school," and "has trouble getting along with teachers." The three item responses were "not," "sometimes," or "often" true. In contrast to Straus et al.,[1] we used a natural log transformation of the 1990 antisocial scale to reduce its skewness, so that the comparisons would not be overly influenced by outliers. Then we scaled the log-transformed 1990 antisocial scale so that it would have the same mean of 50 and the same standard deviation of 20 that was used by Straus et al[1].

The 1988 Antisocial subscale was categorized as being high (top quartile), medium, or low (minimum possible score), as in Straus et al[1]. We also categorized the 1988 log-transformed Antisocial subscale as dichotomous and continuous to determine whether changes in the comprehensiveness of that covariate would influence the size of residual confounding as reflected in increases (for dichotomization) and decreases (for continuous) in the apparent effects of spanking and the nonphysical punishments. To improve the adequacy of the statistical covariate further, we also used the 16-item measure of Externalizing Problems, which included 5-item subscales for Hyperactivity and Headstrong as well as the Antisocial subscale from the Behavior Problems Index[58]. We also used a log transformation to eliminate its skewness. For analyses of simple change scores in the latent factor for Externalizing Behavior Problems, we used the same metric for both waves before calculating gain scores on its three subscales.

As in Straus et al.,[1] spanking was measured by mothers' answers to the question, "About how many times, if any, have you had to spank your child in the past week?" This was immediately followed with parallel questions about how many times, if any, she had " [to] ground him/her," "taken away TV or other privileges," and "sent the child to his/her room" during the past week. Following Straus et al., we combined three or more times into the most frequent category. Finally, to compare the results with another corrective action that a parent might resort to, we used a Yes-No item that asked whether the child had "seen a psychiatrist, psychologist, or counselor about any behavioral, emotional or mental problem during the past 12 months."

Other maternal qualities were measured with the NLSY subscales of cognitive stimulation and emotional support from a short version of the Home Observation for Measurement of the Environment (HOME)[58,59]. Items on the cognitive stimulation scale included maternal reports about reading to the child, taking the child to museums, and the orderliness of the home. Emotional support included items on whether the mother introduced the child by name, kissed or hugged the child, or showed a positive feeling toward the child during the home visit. Following Straus et al., we recalculated the emotional support scale after dropping two of its items about spanking.

Finally, following Straus et al.,[1] socioeconomic status (SES) was measured as the mean of standardized z-scores for (1) the occupational status of the mother's occupation, (2) the total family income, and (3) the mother's educational level. The mean z-score was used even if one of the three components were missing. Ethnicity was coded as ethnic minority or Caucasian, following the original article.

## Results

Table 1 shows the frequencies of the four disciplinary tactics during the week preceding the 1988 interviews and the prevalence of psychotherapy during the previous year. Sending children to their room was the only tactic used more frequently than spanking with these 6- to 9-year-old children.

**Table 1:** Frequencies of Four Disciplinary Tactics and Prevalence of Psychotherapy

Corrective Action	0	1	2	3+
Spanking	449	158	111	67
Grounding	581	109	57	38
Privilege removal	573	117	53	42
Sending to room	389	183	98	115
Psychotherapy^a^	748	34	--	--

^a^Dichotomous, i.e., whether child had seen a psychotherapist in the past year.

Table 2 shows the inter-correlations among the four disciplinary tactics, psychotherapy, continuous measures of antisocial behavior in 1988 and 1990, and other variables used in the structural equation models described later. The frequencies of the disciplinary tactics were moderately correlated with each other (mean *r *= .29 below the diagonal), but less so after dropping children receiving none of these disciplinary tactics in the past week (mean *r *= .15 above the diagonal). A substantial part of the overlap between disciplinary-tactic frequencies is thus due to the 27% of the sample who received none of the four disciplinary tactics in this study.

**Table 2:** Inter-Correlations for Disciplinary Tactics, Psychotherapy, Control Variables, Antisocial Behavior, Hyperactivity, and Headstrong Subscales

Variable		**1**.	**2**.	**3**.	**4**.	**5**.	**6**.	**7**.	**8**.	**9**.	**10**.	**11**.	**12**.	**13**.	**14**.	**15**.	**16**.	**17**.
1. Spank		1.	.14	.09	.09	-.00	-.21	-.12	-.07	-.07	.08	.15	.16	.21	.22	-.03	-.01	.02
2. Grounding		.27	1.	.35	.07	.04	-.13	-.07	-.08	-.04	.18	.20	.14	.18	.22	-.07	-.05	.04
3. Priv. removal		.22	.42	1.	.16	.02	.06	-.06	-.05	-.02	.14	.03	.01	.04	.08	-.03	-.02	.05
4. Sent to room		.31	.22	.30	1.	.03	.10	-.01	.03	.00	-.12	.04	.12	.08	.01	.05	-.02	-.07
5. Therapy		.01	.04	.03	.04	1.	.00	-.04	-.11	.05	-.06	.16	.13	.11	.14	.02	-.01	.03
6. Cogntv stim		-.17	-.11	.04	.07	-.01	1.	.24	.03	.24	-.26	-.13	-.10	-.22	-.23	-.04	.01	-.01
7. SES		-.10	-.07	-.06	-.02	-.02	.22	1.	.02	.14	-.09	-.08	-.01	-.14	-.12	-.05	-.08	.01
8. Female		-.10	-.09	-.08	-.03	-.13	.02	.01	1.	.06	.00	-.13	-.09	-.12	-.16	-.02	.02	-.05
9. Emot. support		-.07	-.05	-.04	-.02	.03	.25	.15	.04	1.	-.16	-.06	-.03	-.10	-.17	-.03	-.01	-.07
10. Non-White		.09	.16	.13	-.06	-.07	-.25	-.07	.05	-.17	1.	-.04	-.16	.05	.08	.03	.03	.03
[1988 Behavior Problem Index subscales]:																		
11. Hyperactivity		.19	.21	.08	.12	.15	-.12	-.08	-.13	-.05	-.02	1.	.55	.44	.39	-.41	-.13	-.04
12. Headstrong		.23	.19	.09	.21	.11	-.07	-.06	-.11	-.04	-.11	.56	1.	.47	.37	-.16	-.41	-.09
13. Antisocial		.27	.22	.11	.18	.10	-.19	-.15	-.15	-.09	.07	.44	.49	1.	.49	-.13	-.15	-.49
[1990 Behavior Problem Index subscale]:																		
14. Antisocial		.27	.25	.14	.13	.12	-.21	-.15	-.17	-.15	.09	.41	.39	.49	1.	.16	.25	.52
[Gain in Behavior Problem Index subscales from 1988 to 1990]:																		
15. Hyperactivity		-.04	-.06	-.04	.02	.00	-.02	-.04	-.01	-.03	.02	-.42	-.15	-.12	.15	1.	.39	.28
16. Headstrong		-.03	-.06	-.03	-.04	.00	.01	-.04	.03	.00	.01	-.13	-.40	-.16	.26	.39	1.	.39
17. Antisocial		.01	.03	.04	-.05	.03	-.02	-.01	-.03	-.06	.02	-.03	-.09	-.49	.53	.26	.41	1.
Full sample statistics																		
Mean		.74	.43	.44	.92	.04	.06	.07	.49	.03	.59	1.68	1.71	50.29	50.17	-.05	.01	-.12
*SD*		.994	.826	.839	1.096	.204	.733	.677	.500	.785	.492	.364	.364	18.99	19.55	.338	.339 19.52	
Subsample statistics																		
Mean		1.02	.59	.61	1.27	.05	.06	.05	.46	.00	.61	1.71	1.76	53.09	52.93	-.06	.00	-.16
*SD*		1.038	.918	.931	1.102	.213	.733	.665	.499	.790	.489	.361	.351	18.84	19.30	.335	.341 19.26	

Note: *N *= 785 below diagonal for full sample (782 for therapy). *N *= 571 above diagonal for subsample with some use of at least one tactic (570 for therapy). Cogntv stim = Cognitive stimulus. *P *< .05 if |r| > .08.

Table 3 shows the re-analysis of Straus et al[1]. Like the original study, antisocial behavior in 1990 was significantly higher for those spanked more in 1988, P < .05, and for boys, P < .05, controlling for antisocial behavior in 1988, P < .001. The results from the re-analysis differed from the original results in the following ways: First, the effect of spanking was significant at only the .05 level, instead of the .01 level[1]. The drop in significance level was due to our log transformation of antisocial behavior in 1990 which reduced its skewness and the influence of extreme outliers. Socioeconomic status and emotional support each predicted lower subsequent antisocial behavior, P < .05, although they were not significant in Straus et al[1]. Two interactions for Spank X Ethnicity and Spank X Gender were significant in the original study but not in our re-analysis.

**Table 3:** Antisocial Behavior in 1990 by Spanking Frequency in 1988 and Six Other Covariates for Children from 6 to 9 Years Old in 1988

Main Effects and Interactions	Degrees of Freedom	Mean Sum of Squares	*F *Ratio
Predictors from 1988			
Main Effects			
Spanking frequency	3	798.4	2.87*
Gender	1	1576.2	5.66*
Cognitive Stimulation	2	383.3	1.38
Emotional Support	2	1271.3	4.56*
Ethnicity	1	0.8	.00
Socioeconomic status	2	1170.6	4.20*
Antisocial behavior (zero, low, high)	2	12460.2	44.72***
2-Way Interactions With Spanking			
Gender	3	386.4	1.39
Cognitive Stimulation	6	183.4	0.66
Emotional Support	6	375.8	1.35
Ethnicity	3	371.8	1.33
Socioeconomic status	6	190.3	0.68
Antisocial behavior	6	115.5	0.42
Residual	741	278.6	

Note. A natural log transformation of 1990 antisocial behavior was used to reduce skewness. The original 807 cases were reduced to *N *= 785 for this analysis due to missing values on grounding, privilege removal, or sending child to room.

*P < .05. ***P < .001.

**Figure 1:**
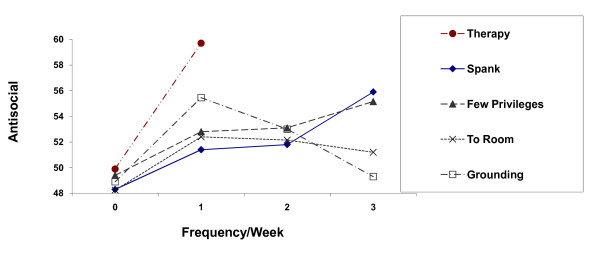
**1990 Antisocial Behavior by Weekly Frequency of Disciplinary Tactics in 1988 (or by Psychotherapy) Controlling for the Same Variables as the Original Study**.

Identical analyses that substituted one of the alternative disciplinary tactics or psychotherapy yielded similar patterns of results (see Figure 1 and Table 4). Grounding and psychotherapy were associated with significantly higher antisocial behavior in 1990 in the parallel analyses, P < .05. Sending children to their room and privilege removal showed similar trends, which was marginally significant for sending children to their room, P < .10, but was not significant for privilege removal. Figure 1 shows the predicted level of antisocial behavior in 1990 for different frequencies of each disciplinary tactic, assuming mean values on all other predictors. Zero use of any disciplinary tactic or psychotherapy was associated with the lowest level of subsequent antisocial behavior. If anything, spanking was associated with lower subsequent antisocial behavior than were alternative disciplinary tactics except when used three times a week or more. None of those differences were significant, however.

**Table 4:** Effects of Alternative Disciplinary Tactics and Psychotherapy in 1988 on Antisocial Behavior in 1990 When Substituted for Spanking in the Analysis Summarized in Table 3

Corrective Action	Degrees of Freedom	Mean Sum of Squares	*F *Value
Spanking	3	798.4	2.87*
Grounding	3	893.1	3.20*
Privilege removal	3	435.4	1.53
Sending to room	3	632.6	2.20^a^
Psychotherapy	1	1116.5	3.93*

*N *= 785, except 782 for Psychotherapy.

^a^*P *< .10.

*P < .05.

Effect sizes were calculated by contrasting the mean antisocial score predicted by zero usage with the weighted mean score predicted by one or more uses of each tactic, because the most debated issue is whether to tolerate any use of spanking whatsoever[60]. The formula for this comparison is: Cohen's , where  is the weighted mean antisocial outcome score predicted by one or more uses of a disciplinary tactic,  is the mean antisocial outcome predicted by zero usage, and *s*_*A *_is the standard deviation for antisocial behavior. Both means were adjusted for all other predictors in the analysis. To compare the effect sizes more directly with the standardized regression coefficients (βs) used to estimate linear associations in the latent-variable analyses, *d *was transformed to an estimated β(β≈*d*/2 for *d*s < .50). *Dstat *was used to calculate the effect sizes from the two means and the mean error sum of squares[61]. Controlling for a trichotomous measure of initial antisocial behavior, the estimated effect sizes were β = .10 for spanking, .12 for grounding, .10 for removing privileges, .09 for sending to room, and .24 for psychotherapy (see Table 5). The result for psychotherapy indicates that this is a general pattern for corrective actions for disruptive behavior problems, whether used by parents or professionals.

**Table 5:** Standardized Regression Coefficients (βs) Predicting 1990 Antisocial Behavior from Four 1988 Disciplinary Tactics and Psychotherapy by Covariate for Pre-Existing Behavior Problems

Covariate for Initial Behavior Problems		Spank	Grounding	Remove Privileges	Send to Room	Psychotherapy
None						
Zero-order *r*		.27*** ^a^	.25***	.14***	.13***	.12**
Controlling for other predictors^b^		.18***	.20***	.11	.14**	.39**
Antisocial Behavior 1988^b^						
Dichotomous		.13**	.17**	.11	.10*	.28*
Trichotomous		.10* ^c^	.12*	.10	.09^f^	.24*
Continuous		.10	.14^f^	.09	.07	.23
Externalizing Behavior 1988^b^						
Continuous (measured variable)		.09	.10	.07	.07	.19
Continuous (latent variable)^d^		.04	.04	.04	-.03	.02
Gain in latent externalizing^e^		-.04	-.07	-.04	-.05	.01

Note. *N *= 785 for the disciplinary tactics; 782 for psychotherapy. A positive β indicates that those receiving the disciplinary tactic in 1988 averaged higher 1990 antisocial behavior than those not receiving that disciplinary tactic, after controlling for the variables indicated. The βs are based on linear associations for the first row and the last 2 rows. The other βs are based on the effect size contrasting the weighted marginal mean antisocial behavior for one or more occurrences of the disciplinary actions (or seeing a psychotherapist) vs. the mean antisocial behavior associated with no occurrences of the disciplinary action or psychotherapy.

^a^This equals the effect size estimated from Straus et al. [1] in Gershoff's meta-analysis,[5] since she based effect sizes on zero-order correlations. (*r *= .27 is equivalent to *d *= .56, using the DSTAT program used by Gershoff)[61]

^b^The "other predictors" were those in the ANCOVA in Straus et al.[1]: gender, ethnicity, SES, cognitive stimulation, emotional support, and the 2-way interactions of the disciplinary tactic with each of those predictors. The 2-way interactions were not included in the structural equation modeling.

^c^Our best re-analysis of Straus et al. [1] Our estimate of β from their graph is .23, but the estimated βs from our 10 samples ranged from .10 to .13, before applying the natural log transformation to 1990 antisocial. In contrast, the *F *values for spanking in our 10 samples ranged from 3.0 to 5.3, which included the original *F *value (4.4) within its range.

^d^The latent structural equation modeling analyses estimate linear associations between the frequency of each corrective action and Antisocial behavior in 1990, controlling for the other predictors in the model, including latent Externalizing problems, which has three indicators (Antisocial, Hyperactivity, and Headstrong), with correlated residuals between the latter two subscales. Fit indices: χ^2^s (13, *N *= 785) from 35.05 to 40.50, *p*s from .0001 to .0008; CFIs from .971 to .976; RMSEAs from .046 to .052.

^e^This row summarizes the only analyses predicting the 16-item Externalizing score in 1990, in this case in terms of gain scores in that latent score from 1988 to 1990 (see Figure 2). Fit indices: χ^2^s (12, *N *= 785) from 7.65 to 13.06, *p*s from .36 to .81; CFIs from .996 to 1.000; RMSEAs from .000 to .011.

^f^*P *< .10.

*P < .05. **P < .01. ***P < .001.

The next set of analyses investigated whether these effect sizes and their significance varied by the adequacy of the measure of initial antisocial behavior. Two overall patterns stand out in Table 5. First, the zero-order correlations indicate that each corrective action, whether by parents or psychotherapists, was significantly associated with higher antisocial behavior two years later, *P*s < .01. Second, all effect sizes decreased in magnitude and changed from apparently detrimental to small, non-significant effects with the addition of increasingly comprehensive measures of pre-existing antisocial behavior (from the top to the bottom of Table 5).

When based on unadjusted correlations, the effect size between spanking and antisocial behavior was identical to the effect size estimated by Gershoff[5] for Straus et al.[1] (i.e., her *d *= .56 corresponds to our *r *= .27 for spanking in the top row of Table 5). The strongest causal evidence against corporal punishment in her meta-analysis was based on this type of unadjusted longitudinal correlation. However, the top row in Table 5 shows that all disciplinary tactics and psychotherapy were also correlated significantly with antisocial behavior two years later, *P*s < .01, although to a smaller degree. Thus, longitudinal correlations do not discriminate between effective and counterproductive corrective actions, but are biased against all corrective actions[20,48].

The association between spanking and subsequent antisocial behavior became smaller and non-significant as the covariate for initial antisocial behavior was measured more comprehensively and reliably, thereby reducing residual confounding. The mean regression coefficient for the other three disciplinary tactics was almost identical to the coefficient for spanking. The standardized coefficients for spanking rarely differed by more than .03 from the parallel coefficient for any alternative disciplinary tactic after initial differences on antisocial behavior were controlled for statistically. Beginning with the third row in Table 5, initial antisocial behavior was statistically controlled with increasingly comprehensive measures. Rows 3 through 6 show that the apparently detrimental effects of disciplinary tactics became nonsignificant when initial antisocial behavior was measured with a continuous measure rather than the less discriminating distinction among low, medium, and high antisocial behavior. The signs of the estimated regression coefficients remained positive for all disciplinary tactics, indicating a nonsignificant tendency for each tactic in 1988 to be associated with higher antisocial behavior in 1990 (see Figure 1).

**Figure 2:**
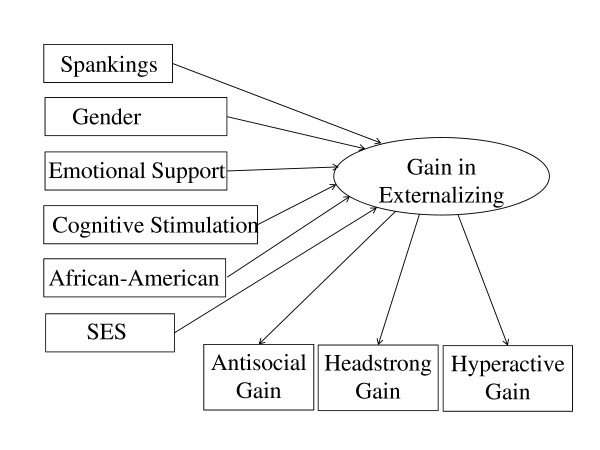
**Structural Equation Model of Latent Gain Scores in Externalizing Behavior Problems from 1988 to 1990**.

The last two rows of Table 5 summarize results from structural equation modeling, controlling in two distinct ways for initial differences on the 16-item measure of externalizing problems. The next-to-last row minimizes measurement error in the covariate by modeling externalizing problems as a latent factor, with three indicators corresponding to the three component subscales (antisocial, headstrong, and hyperactivity). The final row predicts change from 1988 to 1990 in the latent factor for externalizing behavior problems. The indicators for the change score in latent externalizing were gain scores for each of the three component subscales from 1988 to 1990 (see Figure 2).

The structural equation models (SEMs) differed from the other statistically controlled analyses in several ways. First, they predicted linear trends, whereas the other standardized regression coefficients in Table 5 contrasted the mean outcomes for usage vs. non-usage of disciplinary tactics. Second, the SEM analyses did not include any interactions between spanking and the other predictors. Third, the SEM analyses minimized measurement error in the covariate for externalizing problems, which approximates a crucial assumption for making unbiased estimates of causal effects, namely that covariates must be measured without error. Finally, the SEM analyses controlled for the broad measure of externalizing rather than only the 6-item antisocial subscale. It should be noted that only the last SEM analyses of change scores in latent externalizing problems used all 16 items of externalizing behavior problems for the outcome variable in 1990. All other analyses in Table 5 used the 6-item antisocial subscale as the outcome variable, varying only the comprehensiveness with which initial differences in antisocial behavior or externalizing problems were controlled for statistically.

Controlling for the latent externalizing factor in the next-to-last row of Table 5 reduced the effect size further. In these analyses, none of the disciplinary tactics nor psychotherapy was significantly related to antisocial behavior two years later. Moreover, when predicting gain scores in the latent externalizing factor in the last row, the coefficients changed signs so that all disciplinary tactics predicted greater *reductions *in latent externalizing problems, albeit non-significantly so.

### Accounting for Covariations in Use of Tactics

Because mothers who used one disciplinary tactic were more likely to use other tactics, the analyses were repeated in two ways to take that covariation into account. First, all the analyses were repeated for the 73% of the families who reported using at least one of these tactics in the reporting week (see Table 6). Thus these analyses investigated the apparent effects of these disciplinary tactics only for those mothers who saw a need to use at least one of them. The adverse effects of spanking, privilege removal, and sending children to their room then became non-significant after controlling for any measure of pre-existing differences on antisocial behavior. Grounding and psychotherapy showed significantly adverse effects for some measures of initial antisocial behavior, but not when controlling for the more comprehensive measure of externalizing behavior problems.

**Table 6:** Standardized Regression Coefficients (βs) Predicting 1990 Antisocial Behavior from Four 1988 Disciplinary Tactics and Psychotherapy by Covariate for Pre-Existing Behavior Problems After Dropping Cases Using No Disciplinary Tactics of Any Kind

Covariate for Initial Behavior Problems		Spank	Grounding	Remove Privileges	Send to Room	Psychotherapy
None						
Zero-order *r*		.22***	.22***	.08^d^	.01	.14**
Controlling for other predictors^a^		.11^d^	.15***	.04	.03	.38**
Antisocial Behavior 1988^a^						
Dichotomous		.09	.14**	.06	.02	.30*
Trichotomous		.08	.11**	.08	.05	.23
Continuous		.08	.13*	.06	.03	.24
Externalizing Behavior 1988^a^						
Continuous (measured variable)		.08	.08	.05	.05	.24
Continuous (latent variable)^b^		.03	.04	.04	-.05	.02
Gain in Latent Externalizing^c^		-.03	-.07	-.02	-.02	.01

Note. *N *= 571 for the disciplinary tactics; 570 for psychotherapy. A positive β indicates that those receiving the disciplinary tactic in 1988 averaged higher 1990 antisocial behavior than those not receiving that disciplinary tactic, after controlling for the variables indicated. The βs are based on linear associations for the first row and the last 2 rows. The other βs are based on the effect size contrasting the weighted marginal mean antisocial behavior for one or more occurrences of the disciplinary actions (or seeing a psychotherapist) vs. the mean antisocial behavior associated with no occurrences of the disciplinary action or psychotherapy.

^a^The "other predictors" were those in the ANCOVA in Straus et al. [1]: gender, ethnicity, SES, cognitive stimulation, emotional support, and the 2-way interactions of the disciplinary tactic with each of those predictors. The 2-way interactions were not included in the structural equation modeling or the gain in latent externalizing problems.

^b^The latent structural equation modeling analyses estimate linear associations between the frequency of each disciplinary tactic and Antisocial behavior in 1990, controlling for the other predictors in the model, including latent Externalizing problems, which has three indicators (Antisocial, Hyperactivity, and Headstrong), with correlated residuals between the latter two subscales. Fit indices: χ^2^s (13, *N *= 571) from 34.67 to 37.49, *p*s from .0003 to .0010; CFIs from .964 to .968; RMSEAs from .054 to .057.

^c^This row summarizes the only analyses predicting the 16-item Externalizing score in 1990, in this case in terms of gain scores in that latent score from 1988 to 1990 (see Figure 2). Fit indices: χ^2^s (12, *N *= 571) from 10.88 to 15.65, *p*s from .19 to .54; CFIs from .980 to 1.000; RMSEAs from .000 to .024.

^d^*P *< .10.

*P < .05. **P < .01. ***P < .001.

A second way to account for the covariation of disciplinary methods was to include all disciplinary tactics and psychotherapy in the same analyses for the full sample, thereby controlling for each other. Because this greatly expands the number of cells in the ANOVAs or ANCOVAs, this set of analyses did not include any interactions of other covariates with the disciplinary tactics (see Table 7). When controlling for each other, spanking, grounding, and psychotherapy all showed significantly adverse effects, except when controlling for a latent factor for externalizing behavior problems. In contrast, privilege removal and sending children to their room never predicted subsequent antisocial behavior after controlling for the other three corrective actions. The differences in adverse effects by corrective action did not vary as much by covariate comprehensiveness, except when minimizing measurement error by controlling for a latent factor for externalizing behavior problems. This implies that, by controlling for the other corrective actions, the analyses are already controlling for initial child differences, so that adding or improving measures of initial behavior problems does not modify the apparent effects of the corrective actions as much as in the previous analyses. The distinctive pattern of the results in Table 7 is due partly to the tendency for small differences in associations with the outcome to be exaggerated when two correlated predictors are included as predictors in the same multiple regression analysis. [[62], pp. 309-312] When controlling for other disciplinary tactics, corrective actions that parents resort to for more difficult behavior problems appear more detrimental than disciplinary tactics used for milder misbehavior (e.g., grounding vs. sending to room). This suggests that the differences in outcomes among disciplinary tactics may be due to differential selection biases among them. Like psychotherapy, spanking and grounding tend to get selected for more difficult misbehavior than privilege removal or sending children to their room. Differential selection bias would explain why psychotherapy appears as detrimental as spanking and grounding and why these adverse effects became non-significant, sometimes with reversed signs in the latent-variable analyses.

**Table 7:** Standardized Regression Coefficients (βs) Predicting 1990 Antisocial Behavior from Four 1988 Disciplinary Tactics and Psychotherapy by Covariate for Pre-Existing Behavior Problems When Entered Simultaneously (and Without Interactions)

Covariate for Initial Behavior Problems		Spank	Grounding	Remove Privileges	Send to Room	Psychotherapy
None						
Control only for other tactics		.23***	.21***	.02	-.00	.27**
Controlling for other predictors^a^		.19***	.18***	.01	.05	.24**
Antisocial Behavior 1988^a^						
Dichotomous		.15***	.17**	.02	.02	.22*
Trichotomous		.12**	.14*	.01	.01	.19*
Continuous		.12*	.15**	.02	.00	.19*
Externalizing Behavior 1988^a^						
Continuous (measured variable)		.12*	.12^d^	.02	.01	.13
Continuous (latent variable)^b^		.04	.03	.04	-.05	.02
Gain in Latent Externalizing^c^		-.02	-.06	.00	-.03	.02

Note. *N *= 782, except *N *= 785 for the structural equation models. A positive β indicates that those receiving the disciplinary tactic in 1988 averaged higher 1990 antisocial behavior than those not receiving that disciplinary tactic, after controlling for the variables indicated. The βs are based on linear associations for the last 2 rows. The other βs are based on the effect size contrasting the weighted marginal mean antisocial behavior for one or more occurrences of the disciplinary actions (or seeing a psychotherapist) vs. the mean antisocial behavior associated with no occurrences of the disciplinary action or psychotherapy. In contrast to Tables 5 and 6, all results control for the other four corrective actions.

^a^The "other predictors" were those in the ANCOVA in Straus et al.[1]: gender, ethnicity, SES, cognitive stimulation, and emotional support as well as the other four corrective actions. The 2-way interactions were not included in any analyses in this table.

^b^The latent structural equation modeling analysis estimates linear associations between the frequency of each disciplinary tactic and Antisocial behavior in 1990, controlling for the other predictors in the model, including latent Externalizing problems, which has three indicators (Antisocial, Hyperactivity, and Headstrong), with correlated residuals between the latter two subscales. Fit indices: χ^2 ^(22, *N *= 785) = 47.69, *p *= .0008; CFI = .974; RMSEA = .040.

^c^This row summarizes the only analysis predicting the 16-item Externalizing score in 1990, in this case in terms of gain scores in that latent score from 1988 to 1990 (see Figure 2). Fit indices: χ^2 ^(21, *N *= 785) = 22.06, *p *= .34; CFI = .993; RMSEA = .011.

^d^*P *< .10.

*P < .05. **P < .01. ***P < .001.

## Discussion

The present study reanalyzed the strongest causal evidence against customary spanking[1] in order (a) to investigate which alternative forms of discipline would reduce antisocial behavior more than spanking, and (b) to determine whether these apparent causal effects could be attributed to residual confounding due to a selection bias. The results varied somewhat by whether the analyses considered one disciplinary tactic at a time in the full sample, whether the analyses were limited to a misbehaving subsample, or whether the tactics were all included as simultaneous predictors in the analyses. All three types of analyses yielded similar findings when controlling for the most valid and reliable measure of initial differences in externalizing behavior problems, however. The results for the first type of analysis (one disciplinary tactic at a time on the full sample) will be discussed first because it was used in the original study[1]. This will be followed by a brief consideration of the similarities and differences in the alternative types of analyses, which are summarized in Tables 6 and 7.

### Independent Analyses of Disciplinary Tactics in the Full Sample

The first purpose of this study was to compare the child outcomes of spanking with outcomes for alternative disciplinary actions that parents could use instead of spanking. When analyzed one at a time, more frequent use of all three types of nonphysical punishment was associated with higher subsequent antisocial behavior, with effect sizes similar to spanking, as shown in Table 5. No alternative disciplinary tactic was associated with significantly lower antisocial behavior, even after improving the covariate measures. Grounding and psychotherapy were associated with significantly higher antisocial behavior as often as spanking was. Removing privileges and sending children to their room did not have as many significant associations with antisocial behavior, but their effect sizes were similar to those of spanking. The effect size (β) for spanking was within .03 of the mean effect size for the three disciplinary alternatives when controlling for initial antisocial behavior in various ways. Psychotherapy had consistently more adverse effect sizes than spanking and the other disciplinary tactics, except for unadjusted correlations and the first structural equation model (see the next-to-last row of Table 5).

The second purpose of the study was to see whether the apparent causal effects would remain significant after improving the covariate measure of pre-existing antisocial behavior. Consistent with a residual confounding explanation, the apparently adverse effects of all disciplinary tactics and psychotherapy became non-significant as the covariate measure of antisocial behavior became more comprehensive and reliable. The non-significant coefficients changed signs when predicting simple gain scores in a latent externalizing variable in the final analysis, consistent with residual confounding due to selection biases [48,56].

### Analyses of the Subsample Receiving Some Disciplinary Correction

Part of the selection bias occurred because the zero-use group for each disciplinary tactic included the best-behaved children whose behavior never led to any of these disciplinary corrective actions in the referent week. To evaluate the role of this part of the bias, all the analyses were repeated for the subsample that required at least one disciplinary tactic during the referent week (Table 6). After removing the 27% of the children who received no disciplinary tactics, spanking never predicted significantly greater subsequent antisocial behavior after controlling for initial antisocial behavior. Grounding and psychotherapy showed generally more adverse effects than spanking, whereas privilege removal and sending children to their room had smaller non-significant effect sizes than spanking.

### Analyzing All Corrective Actions Simultaneously in the Full Sample

A second alternative to the original type of analysis was to include all disciplinary tactics and psychotherapy as simultaneous predictors in analyses of the full sample. These analyses accentuated the small differences in effect sizes, resulting in near-zero effect sizes for privilege removal and sending children to their room in all of the analyses (see Table 7). In contrast, spanking, grounding, and psychotherapy were associated with significantly higher subsequent antisocial behavior in most of the analyses, except when controlling for the most comprehensive measure of externalizing behavior problems, especially in its more reliable latent form.

Regardless of the type of analysis, all disciplinary tactics and psychotherapy showed small non-significant associations with antisocial behavior when the measure of pre-existing differences maximized comprehensiveness and minimized measurement error in the latent variable analyses in the last two rows of Tables 5, 6, and 7. Moreover, the non-significant associations generally reversed signs when predicting simple gain scores in the latent variable of externalizing behavior problems, consistent with small residual selection biases[44,48,56]. These results suggest that the findings of all three types of analyses are due to residual selection biases that are minimized by controlling for a latent variable for externalizing behavior problems to reduce measurement error and to maximize the comprehensiveness of the proxy for the selection process.

### Differential Selection Biases: Mild vs. Other Corrective Actions

Why do sending children to their room and privilege removal appear to have less adverse effects than spanking, grounding, and psychotherapy when using covariates of intermediate adequacy in the latter two types of analyses (Tables 6 and 7)? The simplest explanation is that the selection bias is smaller for the two mildest disciplinary tactics, creating a differential selection bias in the latter two types of analyses. When each disciplinary tactic is investigated by itself in the full sample, the selection bias is enhanced by well-behaved children requiring no corrective actions in the reporting week. After removing those best-behaved children, the remaining selection bias is minimal for mild disciplinary tactics. Grounding and psychotherapy retained some significantly adverse effects after dropping the best behaved children, probably because they tend to be selected for more problematic misbehavior than are the milder tactics.

Allowing for each disciplinary tactic to be a statistical control for the other disciplinary tactics (and psychotherapy) proved to be similar to controlling for initial antisocial behavior, so that improving the adequacy of the covariate for initial antisocial behavior did not change the effect sizes or significance as much in Table 7 as in the original study's type of analyses in Table 5. In fact, privilege removal and sending children to their room never significantly predicted subsequent antisocial behavior even when controlling only for the other three corrective actions. In one sense, these results illustrate the main point of this study, namely that frequencies of disciplinary tactics are confounded with the behavioral difficulty causing mothers to select those tactics more often. Controlling for the frequency of disciplinary responses to more oppositional misbehavior eliminated the smaller selection bias for the two mildest disciplinary tactics. In contrast, the generally significant effects for spanking, grounding, and psychotherapy indicate that, at any level of using the other tactics, more frequent use of each of those three corrective actions is associated with greater subsequent antisocial behavior than is less use of that corrective action. In other words, given the same degree of behavioral difficulty as indexed by the other disciplinary tactics, greater use of spanking, grounding, and psychotherapy is associated with higher levels of antisocial behavior two years later. But these adverse effects also seem to be due to residual confounding due to selection biases.

There are other indications of a differential selection bias for the two milder tactics compared to the other corrective actions. Sending children to their room, for example, was probably selected for milder behavior problems than was the typical case for grounding. Sending children to their room was the most frequently used tactics, grounding the least used. Sending children to their room had smaller correlations with antisocial behavior in 1990 and 1988 than did grounding, although the difference was smaller for 1988 antisocial behavior. The most important evidence of a differential selection bias is that all significant effects disappeared after controlling for a latent variable for externalizing behavior problems, which maximized the comprehensiveness, validity, and reliability of the covariate.

In sum, the apparently adverse effects of all these disciplinary tactics and psychotherapy seem to be due to selection biases that are stronger for spanking, grounding, and psychotherapy than they are for the two milder disciplinary tactics. Accordingly, the originally adverse effects of spanking replicate for grounding and psychotherapy and are marginally adverse for sending children to their room (Table 5). Across all analyses, grounding and psychotherapy showed as many significantly adverse effects as spanking. The adverse effects of all of these corrective actions became smaller and non-significant when the adequacy of the covariate for pre-existing antisocial behavior was improved.

#### Implications

The general failure of spanking to show more adverse effects than grounding and psychotherapy in our closest re-analyses in Table 5 is remarkable because the original study produced the largest estimate of an adverse causal effect for customary spanking to date[1]. First, the unadjusted longitudinal correlation in this cohort was larger than Gershoff's [5] average for longitudinal studies of corporal punishment and antisocial outcomes (*d *= .56 [*r *= .27] compared to a mean of *d *= .37 [*r *= .18])[63]. Second, this cohort had the largest longitudinal correlation between spanking and subsequent antisocial behavior out of the five NLSY cohorts considered by Straus et al[1]. Third, the Straus et al. study had stronger and more consistent causal evidence against spanking than any of the other six prospective studies that have predicted antisocial behavior from customary spanking of children under the age of 13. Therefore the generally similar outcomes for grounding, psychotherapy, and spanking are not due to selecting a sample with a weak effect for spanking.

These results are all consistent with the conclusion that the apparent effects of all of these corrective actions are due to residual confounding from the tendency of more oppositional children to be selected more often for disciplinary corrective actions and for professional corrective actions. Statistically controlling for pre-existing differences reduces this selection bias confound, but fallible covariate measures do not eliminate it. When initial differences in levels of externalizing behavior problems were measured more comprehensively and reliably with latent variables, no corrective action predicted significantly higher antisocial behavior two years later. This result and the similar pattern of results for all corrective actions by parents and professionals are what would be expected if these results were due to residual confounding with a selection bias. When we changed the direction of the bias by predicting latent change scores, then the apparent effects of all of these disciplinary tactics were not only non-significant, but reversed the signs of their coefficients. This is because the usual analyses of residualized gain scores in net-effects regression is biased against corrective actions, but analyses of simple gain scores are biased in favor of them[48,51,64].

Overall, this is the same pattern of evidence found in a major early evaluation of Head Start,[43] which concluded that the summer version of Head Start was detrimental. Similar to Straus et al.,[1] the Head Start evaluation controlled statistically for the major confound, which was socioeconomic status. Fortunately, Campbell and others recognized what has been illustrated in the present study - that matching and statistical controls are only partially adequate in correcting for this confound - leaving residual confounding, which Campbell called the *under-adjustment bias*[44,65]. Similar to the present study, subsequent re-analyses showed that the apparently detrimental effects of Head Start disappeared with improved covariate measures, but the re-analyses never reversed the effect of summer Head Start into a significantly beneficial effect [66-68].

Other research has shown that most of the corrective actions in this study can be used skillfully to reduce behavior problems in children. Beneficial effects have been demonstrated from more causally conclusive designs for some psychotherapies,[27,28], time out,[69] privilege removal,[70] and spanking when used to enforce time out in clinically defiant 2- to 6-year-olds[23].

In addition to residual confounding, three other methodological aspects of this type of longitudinal analysis may suppress the detection of effective corrective actions. The first aspect that might suppress evidence of effectiveness is that overly frequent use of any disciplinary tactic reflects less effective ways of implementing it. The more effectively any disciplinary tactic is used, the less the child will misbehave and the less often a parent will need to resort to that disciplinary tactic again. Therefore more effective use of any disciplinary tactic will be associated with a lower frequency of using it, other things being equal. This would be particularly true of last-resort tactics, such as spanking or psychotherapy. Frequent use of a last-resort tactic is a symptom of dysfunction in the entire disciplinary system as well as a symptom of the challenge to that system by the child's oppositional behavior.

A second factor that might suppress evidence of effectiveness is that two years is too long an interval to detect a causal effect of the frequency of any disciplinary tactic during one week. Many events may occur in the course of two years that influence antisocial behavior in the life of a child, including genetic effects, peer effects, and other parenting effects. These other causal influences may account for almost all of the development of antisocial behavior over the next two years, leaving little more to be explained by how often disciplinary punishments were used during a single week two years earlier.

A third suppressor of effectiveness might be exclusive reliance on maternal report, which has limited reliability and some likely biases. It is well known that parental reports of child behavior problems have low positive correlations with reports from teachers, children, and observers (e.g., *r*s from .25 to .27), although mothers' and fathers' reports correlate more highly with each other (*r *= .59)[71]. By asking how often each disciplinary tactic was used in the past week, the reports about disciplinary tactics minimize problems of recall and of subjective generalizations. Limiting parental reports to specific behaviors in a very recent time period has been shown to increase the validity of parental reports in other measures[72]. However, the frequency of use in one week may not be typical of other weeks. In addition, there is some evidence that reliance on maternal reports for all data tends to inflate the evidence of adverse effects of all disciplinary punishments[73].

Finally, the near-zero effects may represent the average of effective and ineffective use of these disciplinary tactics in reducing antisocial behavior. The failure to find between-tactic differences in effectiveness raises the possibility that within-tactic differences in how and when a disciplinary tactic is used may be more important than which tactic is used. From this perspective, different ways that parents use these forms of punishment may counterbalance each other, yielding the overall non-significant coefficient. This view is consistent with anecdotes from behavioral parent trainers, who train parents how to use time out consistently, even though many referred parents say they have tried time out previously without success.

Consistent with this last possibility, the meta-analysis by Larzelere and Kuhn[9] found that the outcomes of physical punishment compared differently with outcomes of alternative disciplinary tactics depending upon how physical punishment was used. Child outcomes of physical punishment compared unfavorably with alternatives only when it was used too severely or as the primary discipline method. The outcomes of customary physical punishment (e.g., spanking frequency) were equivalent to those of alternative disciplinary tactics, consistent with the closest replication of Straus et al.[1] in this study. The meta-analysis also found that spanking could be more effective than alternatives when it was used nonabusively to back up milder disciplinary tactics when 2- to 6-year-olds defiantly refused to cooperate with them. Such back-up spanking led to greater reductions in defiance or antisocial behavior than 10 of 13 alternatives it had been compared with directly. One advantage of back-up spanking is that it enhances the subsequent effectiveness of milder disciplinary tactics, such as time out, so that spanking can be phased out in a matter of weeks[23].

Future research needs to discriminate between effective and counterproductive ways of implementing all disciplinary tactics, so that advice to parents can recommend the mildest effective disciplinary tactics for each situation. Statistically controlled studies of the outcomes of frequency of usage fail to provide those discriminations because frequency measures include no information about how disciplinary tactics were implemented or the disciplinary situations for which they were used.

#### Limitations

Some limitations of this study should be noted. First, all the data were based on maternal report, identical to the original study by Straus et al.[1] and all other statistically controlled longitudinal studies with consistent evidence against customary spanking. It has been shown that evidence based on a single source of information is biased against disciplinary tactics[73]. Second, this study had no data on disciplinary tactics used by fathers, which was also a limitation in the original study.

A third limitation is that we have not duplicated the original study exactly, although we re-analyzed it as closely as possible. In contrast to the original study, we used a log transformation for the antisocial behavior to reduce the influence of extreme outliers in its skewed distribution. We also dropped 22 cases (2.7%) because they had missing data on one or more of the nonphysical consequences in order to ensure that the comparisons among disciplinary tactics were based on exactly the same sample and types of analyses.

## Conclusions

Notwithstanding these limitations, the present study has shown that the strongest causal evidence against customary spanking seems to be due to residual confounding because behaviorally difficult children cause parents to use all disciplinary corrective actions more frequently. When the measure of initial child differences is improved, the evidence against customary spanking becomes non-significant, as does similar evidence against grounding and psychotherapy. The apparently adverse effects become nonsignificant more readily for the two milder disciplinary tactics, most likely because of a differential selection bias, because they tend to be selected more often for milder types of misbehavior than the other corrective actions.

What are the implications for pediatric advice about disciplinary guidance for parents of young children? The disciplinary goal of parents should be to rely as much as possible on the mildest disciplinary response that will be effective for maintaining age-appropriate levels of cooperation. Verbal correction and reasoning can be effective for children as young as two years of age, especially if it is used emphatically and backed up with nonphysical consequences when necessary[21,74]. Milder disciplinary consequences, such as sending children to their room or the more precise version of time out taught by psychologists, have been consistently recommended for young children. Time out is a key component of the most scientifically established parenting treatments for young children with oppositional defiant disorder, conduct disorder, or attention deficit hyperactivity disorder[27,28,75]. The only use of spanking that has been demonstrated to be more effective than alternatives is when it is used to enforce time out when 2- to 6-year-olds refuse to comply with it[9]. Parents or pediatricians who are opposed to the spank back-up should be aware of the one-minute forced room isolation, which is only alternative shown to be as effective as the spank back-up for enforcing time out on a chair[23]. Other enforcements for time out have also been used, but no other enforcement has been shown to be as effective as the above two back-ups for time out.

Even though our results do not show spanking to be causally linked to subsequent antisocial behavior, they should not be understood as an unqualified endorsement of spanking. In the larger debate on whether or not governments should prohibit a parent's right to retain spanking as one disciplinary option, other factors outside of the scope of the present study should be considered, such as relevant moral values and when they are sufficiently compelling to impose non-spanking values on all parents. Parents should choose from the mildest disciplinary tactics that will be effective in any disciplinary situation, but defiant children need stronger tactics to enforce milder tactics to achieve that goal[23].

Although non-empirical considerations may support a ban on spanking, the present study suggests that the strongest causal evidence against non-abusive spanking relies on methods that are inadequate for supporting a general prohibition against spanking or for identifying alternative disciplinary tactics that parents should use instead. Future studies need to use improved research methods to discriminate between effective and counterproductive disciplinary enforcements of all types to provide a stronger scientific basis for disciplinary recommendations, whether parents choose to include spanking as one of their disciplinary options or not.

## List of Abbreviations Used

NLSY: National Longitudinal Survey of Youth; HOME: Home Observation for Measurement of the Environment; SES: Socio-Economic Status; SEM: Structural Equation Modeling.

## Competing interests

The authors declare that they have no competing interests.

## Authors' contributions

REL planned, designed, and implemented the study. RBC had a major role in working with REL in revising and finalizing the manuscript. GLS did most of the initial analyses under the supervision of REL. The final manuscript was read and approved by all authors.

## Authors' information

REL is Associate Professor in the Dept. of Human Development and Family Science (HDFS) at Oklahoma State University. He was a member of the recent Task Force on Physical Punishment of the Child Maltreatment section of Division 37 of the American Psychological Association. RBC is an Assistant Professor in the Dept. of HDFS at Oklahoma State University. GLS is a Senior Research Analyst at Girls and Boys Town, Boys Town, NE, USA.

## Pre-publication history

The pre-publication history for this paper can be accessed here:

http://www.biomedcentral.com/1471-2431/10/10/prepub
